# Alternation of regional homogeneity in trigeminal neuralgia after percutaneous radiofrequency thermocoagulation: A resting state fMRI study

**DOI:** 10.1097/MD.0000000000005193

**Published:** 2016-10-21

**Authors:** Zhi Dou, Xueyi Zhang, Liqiang Yang, Wanqian Wang, Na Li, Zhicheng Liu, Jiaxiang Ni

**Affiliations:** a Department of Pain Management, Xuanwu Hospital Capital Medical University; b School of Biomedical Engineering, Capital Medical University; c Department of Radiology, Xuanwu Hospital Capital Medical University, Beijing, China.

**Keywords:** percutaneous radiofrequency thermocoagulation, regional homogeneity., resting-state fMRI, trigeminal neuralgia

## Abstract

We used resting-state fMRI to investigate regional homogeneity (ReHo) changes in patients with TN before and after PRT procedure, and to speculate about its possible mechanisms.

Thirty-one TN patients underwent the PRT procedure had MRI scans just before and 6 months after surgery. The anatomical and resting-state functional images were all acquired. Patients’ visual analog scales (VAS) scores, facial numbness, and disease duration were also recorded. Voxel-wise ReHo analysis was performed to detect the altered regional clusters after surgery. The correlations between the mean ReHo values of each significant cluster and clinical variables were examined.

Compared with presurgical condition, patients after the PRT procedure showed a significant ReHo value increases in the right fusiform gyrus (FG) and bilateral anterior cingulate cortex (ACC), but decreases in the left inferior parietal lobule (IPL), right calcarine, right middle temporal gyrus (MTG), left postcentral gyrus (PoCG), and left insula. We demonstrated a positive correlation between ReHo in the left PoCG and VAS scores, a negative correlation between pre-surgical ReHo in the right MTG and VAS changes (ΔVAS).

Alterations of ReHo post-surgical were found in several regions, which are related to sensory, affective, and emotional processes. The MTG may be a specific area that is associated with analgesic efficiency of PRT procedure.

## 1 Introduction

TN, the most prevalent disease of facial pain, is characterized by excruciating shooting pain in regions dominated by trigeminal nerve. Despite advances in the therapy of trigeminal neuralgia (TN), such as microvascular decompression (MVD) Gamma Knife radiosurgery (GKRS) and percutaneous radiofrequency thermocoagulation (PRT), some 5% to 10% of patients may not achieve immediate pain relief after initial surgeries, and recurrence rates rise up to 50% at 10 years, leading to significant personal, social, and economic burden.^[1,2]^ Development of more effective treatment strategies for this neuropathic pain disorder will require better knowledge of its neurobiological underpinnings.

According to current opinion, TN is caused by a proximal nuerovascular compression at the trigeminal nerve root entry zone in the prepontine cistern leading to secondary demyelination of trigeminal nerve root.^[3]^ However, it has been evidenced that the compressed nerve may represent a risk factor for the development of TN, but other reasons may also involved in the elicitation of TN.^[4,5]^ Jannetta^[6]^ described 12% of his investigated TN patients had no nerve vessel conflict, whereas as Kakizawa et al^[7]^ indicated that up to 49% of people without TN show a nerve vessel contact on magnetic resonance imaging (MRI). Moreover, numerous MRI studies indicate that both structural and functional alterations of central nervous system exist in patients with TN.^[4,8–10]^

Obermann, using voxel-based morphometry, found that gray matter (GM) volume reduction in TN patients compared to healthy controls in multiple cortical and subcortical areas of the brain and the reduction within anterior cingulate cortex (ACC), parahippocampus, and temporal lobe are correlated with increasing disease duration.^[4]^ By using task functional MRI (fMRI), Moisset et al^[9]^ detected a mass of abnormal activation areas of the brain in TN patients when painful stimuli was applied to the trigger zone.

In resting-state fMRI (rs-fMRI) studies, altered patterns of ReHo were detected in patients with TN. Wang et al^[11]^ reported that the ReHo was decreased in amygdala, parahippocampal gyrus, and cerebellum and increased in the inferior parietal lobule (IPL), inferior temporal gyrus (ITG), postcentral gyrus (PoCG), and thalamus. ReHo measures the local synchronization of spontaneous fMRI signals by calculating similarity of dynamic fluctuations of voxels within a given cluster, which has been recognized as a highly sensitive, reproducible, and reliable neuroimaging marker in diagnosis and assessment of numerous nervous system diseases.^[12,13]^ However, little is known about the alteration of ReHo in TN patients after effective neurosurgical treatment.

Our objective was to detect the whole brain resting-state ReHo changes in patients with TN before and after the PRT procedure in order to identify specific spontaneous activity patterns that may be associated with the development and persistence of this debilitating facial pain condition. We also performed correlation analysis between ReHo and clinical variables so as to speculate about the possible brain areas that mediate the surgical impact on pain relief or facial numbness severity.

## 2 Materials and methods

### 2.1 Patients and study design

In this study, 38 patients who underwent the PRT procedure for classical TN were recruited from the Department of Pain Management at XuanWu Hospital between June 2015 and January 2016. The patients were all right-handed. All of them had right-sided pain affecting the maxillary and/or mandibular division of the trigeminal nerve, and met the criteria for classical TN as outlined by the International Classification of Headache Disorders-II (2004). They are all characterized by little or no pain at rest, with intermittent paroxysms of shooting pain. The exclusion criteria were (1) patients with chronic pain other than TN, (2) patients with neural diseases, diabetes, or brain surgery history, and (3) patients unable to undergo MRI scanning because of claustrophobia or other psychological disorders, or metal implantation.

There were 2 scheduled scanning sessions; 1 was in 1 week before the PRT procedure, and the other one was 6 months after the surgery. Patients on carbamazepine or other pain medications were asked to discontinue use of their medication for 1 week before their MRI scans. To make sure that patients maintained pain-free state during scanning session, they could halt the experiment by activating a safety mechanism held in one hand, if they suffered a facial pain paroxysm.

The duration of pain, frequency of pain paroxysms, and visual analog scales (VAS) for pain intensity were obtained before the first MRI scanning. Facial numbness classification (grade I to IV) was recorded before the second MRI scanning.^[2]^ If any patient got pain recurrence or did not receive pain relief during the following 6 months after the PRT procedure, the VAS would also be recorded. The Research Ethics Committee of Xuanwu Hospital approved this study and written informed consent was obtained from each participant.

### 2.2 PRT procedure

The PRT procedure was performed according to our previously reported.^[2,14]^ The puncture of gasserian ganglion was according to the Hartel anterior route. The best puncture approach to the oval foreman and the corresponding skin insertion point was determined by CT scanning. Motor (2 Hz, 1 ms) and sensory (50 Hz, 0.1 ms) were performed to confirm or readjust the needle tip position to confirm the accuracy. The gasserian ganglion was thermally coagulated with radiofrequency at 75 °C for 120 s.

### 2.3 MRI data acquisition

MRI examinations were acquired using a Siemens Tim Trio 3 T MRI system (Siemens, Erlangen, Germany). Foam padding and headphones were used to limit head motion and reduce scanner noise.^[15]^ All patients were instructed to lie with their eyes open, think of nothing in particular, and not fall asleep. High-resolution T1-weighted anatomical images were obtained by using rapid gradient echo sequence with the following parameters: repetition time (TR) = 1900 ms, echo time (TE) = 2.19 ms, flip angle (FA) = 9°, field of view (FOV) = 256 mm, sections = 176, voxel size = 1 × 1 × 1 mm^3^. Resting-state functional datasets were recorded using a T2∗-weighted echo-planar imaging (EPI) sequence with the following parameters: TR = 2000 ms, TE = 30 ms, FA = 90°, FOV = 240 mm, sections = 33, thickness = 4 mm.

### 2.4 Data processing

All preprocessing steps and ReHo analyses were carried out using statistical parametric mapping (SPM12, http://www.fil.ion.ucl.ac.uk/spm) and Data Processing & Analysis for Resting-state Brain Imaging (DPABI Version 2.1, http://www.restfmri.net).^[16]^ The first 10 volumes of each functional time series were discarded because of instability of initial magnetic resonance imaging signal and adaptation of participants to the circumstance. The remaining 230 volumes were corrected for the acquisition time delay between the different slices and were also corrected for geometrical displacements according to the estimated head movement and were realigned to the first volume. Patients whose head motion translation values were >2.0 mm in any direction of *x*, *y*, *z*, rotation values >2° of any angle, or the mean frame-wise displacement (meanFD) >0.4 mm were excluded.^[17,18]^ After motion correction, the mean structural image was co-registered to the mean realigned functional image and then spatially normalized by using DARTEL (diffeomorphic anatomical registration using exponentiated lie algebra).^[19]^ A multiple linear regression analysis was performed to remove several nuisance signals, including 6 head motion parameters, the white matter signal, and the cerebrospinal fluid signal. The linear trend of the time series was removed and band-pass filtering (0.01 Hz < *f* < 0.1 Hz) was performed to reduce the influence of physiological noise, such as the respiratory and cardiac rhythms. Besides excessive head motion, all patients with bad brain extraction, tissue segmentation and with bad surface construction were also excluded from the subsequent analysis.

### 2.5 ReHo analysis

ReHo is computed as Kendall's coefficient of concordance (KCC) value of the ranked time series of a given voxel to its nearest neighbors. To optimize the trade-off between mitigation of partial volume effects and generation of Gaussian random fields, we chose 27 voxels to calculate the ReHo, as it is more appropriate for covering all directions in 3D space.^[13]^ We implemented the computation of ReHo in individual native spaces to avoid multiple confounding sources introduced by head motion, imperfect image registration, and non-neural physiological processes.^[13]^ For standardization purposes, individual ReHo maps were divided by their global average within the whole-brain mask. Then, spatial smoothing was performed using a 4 mm full-width at half-maximum (FWHM) Gaussian kernel.

### 2.6 Statistical analysis

Within-subject comparisons were made to compare pre- and postsurgical voxel-wise ReHo values using paired samples *t* tests. Voxels with a *P* value < 0.005 and cluster size > 54 were considered to show significant difference, which was equal to a corrected threshold of *P* < 0.05, determined by the Monte Carlo stimulation (the AlphaSim program in the DPABI toolbox).

The correlations between the ReHo values (*z* stats) of these significant clusters and clinical scales in patients were performed by Pearson's correlation analysis with a threshold of *P* < 0.05. The change of ReHo between the pre- and postsurgical conditions (ΔReHo) was also calculated as the correlation coefficient with clinical variables.

## 3 Results

### 3.1 Patients’ characteristics and clinical assessments

For a total of 38 recruited patients, 32 patients completed the 2 scheduled MRI scanning sessions, and other 6 patients were excluded because they refused to receive re-text MRI scanning and lost to follow-up or pain paroxysm during the MRI scanning. One patient was excluded for excessive head motion. Finally, 31 patients were included in subsequent data analysis.

Among patients who completed the whole study, 29 patients achieved immediate excellent pain relief and 2 patients achieved moderate pain relief after the PRT procedure, and no patient had pain recurrence during follow-up. Compared with presurgery condition, the VAS score and pain frequency reduced significantly after the PRT procedure. Moreover, all patients had different degrees of facial numbness after the PRT procedure. Patient demographic and clinical details are presented in Tables 1 and 2.

**Table 1:**
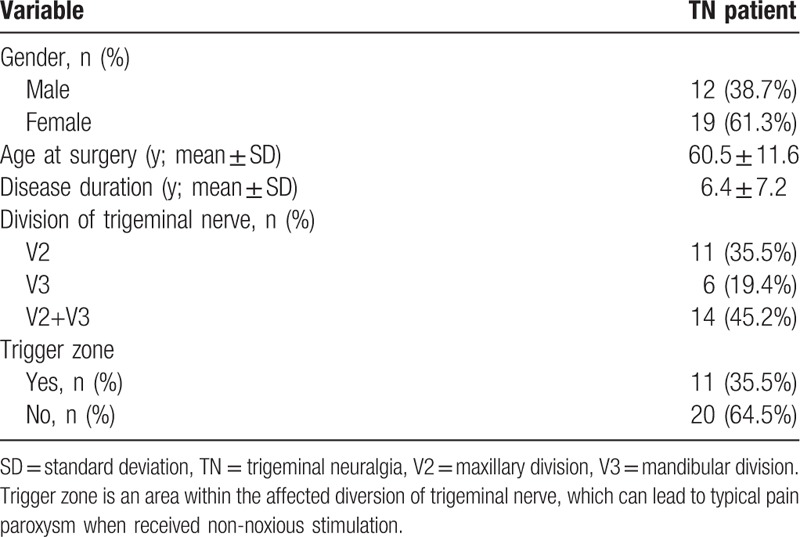
Patient characteristics prior to the study.

**Table 2:**
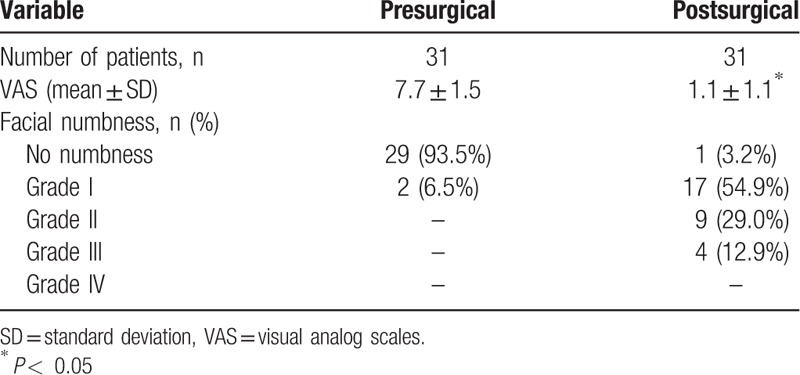
Changes in pain and facial numbness between pre- and postsurgical conditions.

### 3.2 ReHo alterations

**Figure 1:**
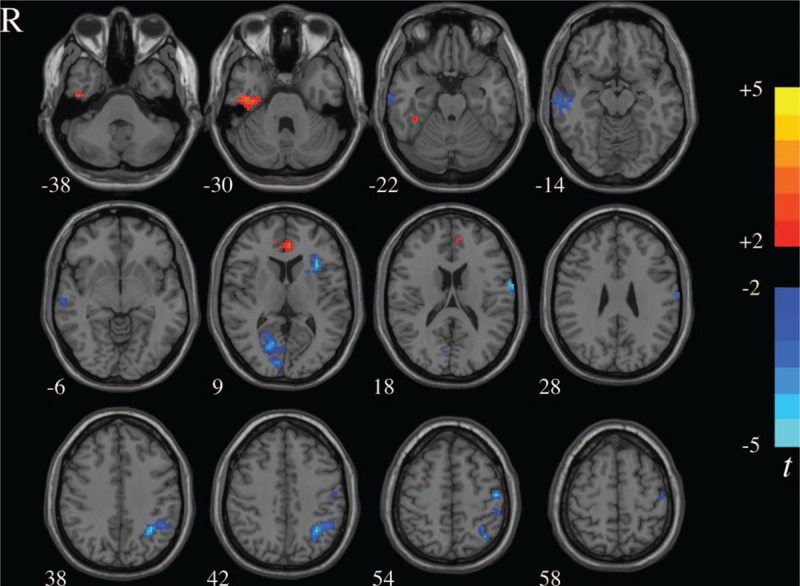
ReHo differences between pre- and postsurgical conditions. The color bar represents the *t* value of the paired *t* test of ReHo. ReHo = regional homogeneity.

In the rs-fMRI images, TN patients showed that significant ReHo value increases in the right fusiform gyrus (FG) and bilateral ACC (*P* < 0.05, AlphaSim corrected), but decreases in the left IPL, right calcarine, right middle temporal gyrus (MTG), left PoCG, and left insula (*P* < 0.05, AlphaSim corrected) after the PRT procedure (Table 3 and Fig. 1).

**Table 3:**
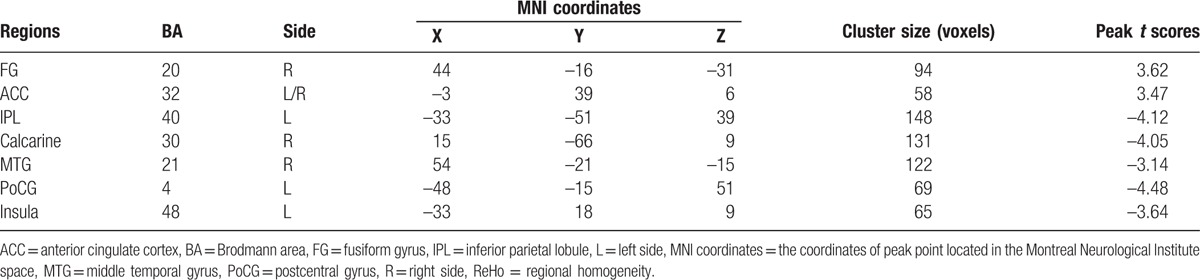
Brain regions with significant different ReHo values pre- and postsurgical (*P* < 0.05, Alphasim corrected).

### 3.3 Correlations between ReHo and clinical variables

**Figure 2:**
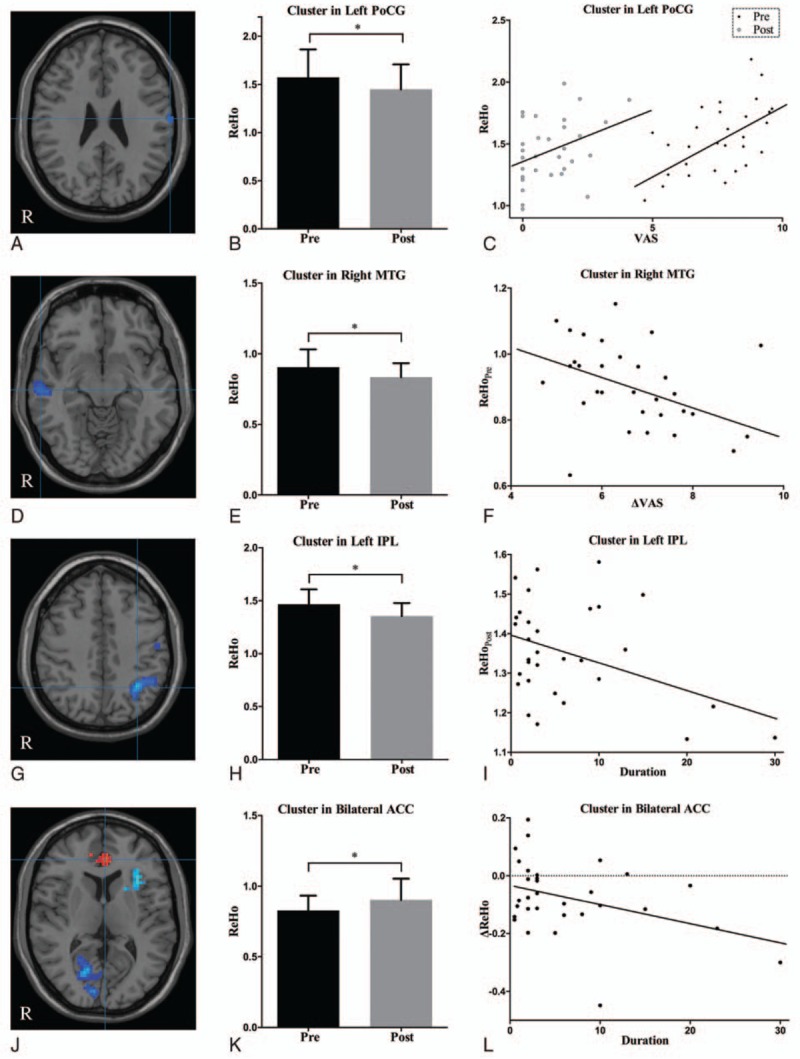
Brain areas that demonstrated altered ReHo between pre- and postsurgical conditions and correlated with clinical variables. (A) Cluster in the left PoCG; (D) cluster in the right MTG; (G) cluster in the left IPL; (J) cluster in the bilateral ACC; (B, E, H, K) ReHo between pre- and postsurgical conditions; (C, F, I, L) ReHo correlated with clinical variables. ^∗^*P* < 0.05. ACC = anterior cingulate cortex, IPL = inferior parietal lobule, MTG = middle temporal gyrus, PoCG = postcentral gyrus, post = post-surgical condition, pre = pre-surgical condition, ReHo = regional homogeneity.

The significantly positive correlation was found between ReHo in the left PoCG and VAS scores (presurgical: *r* = 0.620, *P* <  0.001; postsurgical: *r* = 0.379, *P* <  0.05). The presurgical ReHo in the right MTG was negatively correlated with ΔVAS scores (*r* = –0.360, *P* <  0.05). A significant negative correlation was shown between disease duration and postsurgical ReHo in the left IPL (*r* = –0.358, *P* <  0.05), accompanied by a negative correlation between duration and ΔReHo in the bilateral ACC (*r* = –0.384, *P* <  0.05). The significant correlation between age, numbness, and ReHo was not found (Fig. 2).

## 4 Discussion

This study is the first to explore the alternations of whole brain ReHo in TN patients after the PRT procedure. The current study revealed significantly altered intrinsic resting-state ReHo in several brain regions in patients with TN between pre- and postsurgical conditions.

The involvement of S1, located in the PoCG, in pain processing has been known for a long time.^[20]^ fMRI studies have found that PoCG is activated during noxious stimulation as well as in association with pathological pain states such as TN, postherpetic neuralgia, and chronic low back pain.^[8,20–22]^ In our study, the dramatic decrease of ReHo values at PoCG (especially the area corresponding to the face) after the PRT procedure indicates that PoCG plays a highly modulated role in the sensory aspects, including the localization and discrimination of pain.

Functional studies showed that the insula processes both in acute and chronic pain perception.^[23]^ Borsook et al^[8]^ described an increased activation of the insula during evoked and spontaneous tics in TN patients in comparison to healthy controls. Direct stimulation of the insula in humans can produce intense shock-like pain, which is similar to the paroxysms of TN.^[24]^ In patients with and without allodynia, light tactile stimulation of the affecting division induced activation of the insula. However, the enhanced activation was disappearing after the PRT procedure.^[9]^ The increased activation in the insula may be correlated with the exaggerated expectancy of pain and attention. A recent combined structural and FC analysis of insular subdivisions supported that the anterior insular part is associated with cognitive-affective dimension of pain.^[25]^ According to the same insular subdivision system, we found decreased ReHo in the left anterior insular part in patients after surgery.

Another important area for the occurrence of TN and that shares important interactions with the insula is the ACC. ACC and anterior insula are known to be important parts of salience network, which activates during salient changes in the sensory environment.^[26]^ The rostral ACC and insula are activated by attention to the unpleasantness of pain, and patients with chronic pain exhibit increased functional connectivity between the ACC and anterior insula at rest and during pain processing.^[27,28]^ However, the effect of ACC in TN is still controversial. A large portion of the ACC was active during spontaneous tics in patients with TN.^[8]^ The painful stimulation at the trigger zone activated the bilateral posterior part of ACC [caudal areas of BA (Brodmann area) 24 and 32].^[9,29]^ But the abnormal activity of ACC has not been detected in TN patients at resting state.^[11]^ Of note, we found the decreased rostral ACC (anterior area of BA 32) activation after surgery, which may correspond with its activation in patients with treated complex regional pain syndrome (CRPS) in Becerra's study.^[30]^ These findings suggest that different regions of ACC might play different roles in TN.

IPL and MTG are located at the posterior lateral cortices, which is 1 of 3 major subdivisions of the default mode network (DMN).^[31,32]^ DMN is the most thoroughly investigated and stable resting-state network, and the posterior lateral subdivision of this network is involved in mediating the recognition and rumination of pain.^[26,33]^ Moreover, this network can be affected by a chronic pain state. Greater clinical pain in chronic low back pain and fibromyalgia patients was associated with greater intrinsic DMN and DMN-insula connectivity.^[34]^ In comparison to healthy control, patients with migraine showed decreased connectivity in prefrontal and temporal gyrus of the DMN.^[35]^ Greater connectivity between the DMN and the insula is directly associated with the duration of migraine attacks.^[36]^ In our present work, the decreased activation of both IPL and MTG in patients after surgery was detected. However, the alternations of functional connectivity between the 2 cortices and within the DMN remain to be explored.

A significant ReHo increase was noticed in the right FG, an area often associated with facial recognition.^[37]^ It has been known that this area is involved in both detection and identification of faces.^[38]^ In this study, we did not perform any visual stimulation to the patients, so we inferred that TN might impair the self-face perception of the patients. When pain was relief, the activation of FG was reinforced. This finding is in line with a voxel-based morphometry study on TN. Parise et al^[39]^ found the reduction of cortical thickness at FG in TN, and the thickness is correlated negatively with the carbamazepine dose.

Other than the involvement of facial perception, FG also plays some role in chronic pain condition. Postoperative pain can activate the ipsilateral FG.^[40]^ Patients with low back pain displayed activation of FG, and the activation can be enhanced by visualization of painful experiences.^[41]^ Both structural and functional abnormalities of FG have been identified in patients with migraine. The left FG had an increased volume in patients with aura compared to patients without aura and healthy controls, whereas it was significantly atrophied in patients without aura.^[42]^ The fALFFs (fractional amplitude of low-frequency fluctuation), measures that quantify the activation of brain regions, in migraine patients were significantly decreased in the right FG.^[43]^ However, the assignment of FG in TN in addition to facial perception has not been known at all.

It is worth noting that ReHo is not merely a reliable metric to investigate the spontaneous neuronal activities in resting-state fMRI, but also has correlation with some clinical variables in chronic pain situations.^[34,44,45]^ A novel finding in the present study was the lower baseline ReHo of the MTG was associated with greater pain relief (ΔVAS) after the PRT procedure. The result indicates that the pre-surgical ReHo values in this area can be used as neuroimaging markers for predicting response to the treatment. Moreover, the dynamic ReHo changes observed at PoCG, in association with the varying VAS scores observed in our study, might represent the important role that PoCG plays in monitoring pain intensities. Other findings like the negative correlation between ΔReHo in the bilateral ACC and disease duration, and the negative correlation between post-surgical ReHo in the left IPL and duration, suggested that the dysfunction in these brain regions becomes more and more serious along with TN progression.

There were several limitations that should caution in the interpretation of the current results. First, our results were limited to a small sample size, and no resistant or recurrent patient was observed. So we were not able to apply a subgroup analysis to identify specific brain regions as prognostic indicators. Second, we only examined patients with right-sided pain to avoid flipping the scans. However, it may have produced bias in identification of the predominant side of brain regions. At last, ReHo is a widely used tool for local connectivity analysis, but the remote or long-distance relationships in cerebral cortex parcellation should also be detected.

## 5 Conclusion

Our findings contribute to the growing body of evidence indicating that functional alterations of central nervous system exist in TN patients after receiving the PRT procedure.^[9]^ Alterations of ReHo post-surgical were found in several regions, including PoCG (S1), insula, ACC, IPL, MTG, FG and calcarine, which are related to sensory, affective, and emotional processes. Furthermore, a negative relationship between pain relief and pre-surgical ReHo in the MTG was identified. These results may help to improve our understanding of abnormal neural activities in TN. However, further studies are needed with a large sample size, longitudinal observation, and multimodality imaging analysis.

## References

[1] Gunther T Gerganov VM Stieglitz L Microvascular decompression for trigeminal neuralgia in the elderly: long-term treatment outcome and comparison with younger patients. *Neurosurgery* 2009; 65:477–482.discussion 482. 19687692 10.1227/01.NEU.0000350859.27751.90

[2] Tang YZ Jin D Bian JJ Long-term outcome of computed tomography-guided percutaneous radiofrequency thermocoagulation for classic trigeminal neuralgia patients older than 70 years. *J Craniofac Surg* 2014; 25:1292–1295. 25006910 10.1097/SCS.0000000000000591

[3] Marinkovic S Todorovic V Gibo H The trigeminal vasculature pathology in patients with neuralgia. *Headache* 2007; 47:1334–1339. 17927650 10.1111/j.1526-4610.2007.00933.x

[4] Obermann M Rodriguez-Raecke R Naegel S Gray matter volume reduction reflects chronic pain in trigeminal neuralgia. *Neuroimage* 2013; 74:352–358. 23485849 10.1016/j.neuroimage.2013.02.029

[5] Montano N Conforti G Di Bonaventura R Advances in diagnosis and treatment of trigeminal neuralgia. *Ther Clin Risk Manag* 2015; 11:289–299. 25750533 10.2147/TCRM.S37592 PMC4348120

[6] Jannetta PJ Arterial compression of the trigeminal nerve at the pons in patients with trigeminal neuralgia. *J Neurosurg* 1967; 26:159–162. 10.3171/jns.1967.26.1part2.0159 6018932

[7] Kakizawa Y Seguchi T Kodama K Anatomical study of the trigeminal and facial cranial nerves with the aid of 3.0-tesla magnetic resonance imaging. *J Neurosurg* 2008; 108:483–490. 18312095 10.3171/JNS/2008/108/3/0483

[8] Borsook D Moulton EA Pendse G Comparison of evoked vs. spontaneous tics in a patient with trigeminal neuralgia (tic doloureux). *Mol Pain* 2007; 3:34. 17983481 10.1186/1744-8069-3-34 PMC2217520

[9] Moisset X Villain N Ducreux D Functional brain imaging of trigeminal neuralgia. *Eur J Pain* 2011; 15:124–131. 20609605 10.1016/j.ejpain.2010.06.006

[10] DeSouza DD Davis KD Hodaie M Reversal of insular and microstructural nerve abnormalities following effective surgical treatment for trigeminal neuralgia. *Pain* 2015; 156:1112–1123. 25782366 10.1097/j.pain.0000000000000156

[11] Wang Y Zhang X Guan Q Altered regional homogeneity of spontaneous brain activity in idiopathic trigeminal neuralgia. *Neuropsychiatr Dis Treat* 2015; 11:2659–2666. 26508861 10.2147/NDT.S94877 PMC4610767

[12] Zang Y Jiang T Lu Y Regional homogeneity approach to fMRI data analysis. *Neuroimage* 2004; 22:394–400. 15110032 10.1016/j.neuroimage.2003.12.030

[13] Jiang L Zuo XN Regional homogeneity: a multimodal, multiscale neuroimaging marker of the human connectome. *Neuroscientist* 2015; 22:486–505. 26170004 10.1177/1073858415595004 PMC5021216

[14] Tang YZ Jin D Li XY Repeated CT-guided percutaneous radiofrequency thermocoagulation for recurrent trigeminal neuralgia. *Eur Neurol* 2014; 72:54–59. 24853911 10.1159/000357868

[15] Li Y Liang P Jia X Abnormal regional homogeneity in Parkinson's disease: a resting state fMRI study. *Clin Radiol* 2016; 71:e28–e34. 26628410 10.1016/j.crad.2015.10.006

[16] Yan CG Wang XD Zuo XN DPABI: data processing & analysis for (resting-state) brain imaging. *Neuroinformatics* 2016; 14:339–351. 27075850 10.1007/s12021-016-9299-4

[17] Power JD Barnes KA Snyder AZ Spurious but systematic correlations in functional connectivity MRI networks arise from subject motion. *Neuroimage* 2012; 59:2142–2154. 22019881 10.1016/j.neuroimage.2011.10.018 PMC3254728

[18] Yan CG Cheung B Kelly C A comprehensive assessment of regional variation in the impact of head micromovements on functional connectomics. *Neuroimage* 2013; 76:183–201. 23499792 10.1016/j.neuroimage.2013.03.004 PMC3896129

[19] Ashburner J A fast diffeomorphic image registration algorithm. *Neuroimage* 2007; 38:95–113. 17761438 10.1016/j.neuroimage.2007.07.007

[20] Kong J Spaeth RB Wey HY S1 is associated with chronic low back pain: a functional and structural MRI study. *Mol Pain* 2013; 9:43. 23965184 10.1186/1744-8069-9-43 PMC3765748

[21] Becerra L Morris S Bazes S Trigeminal neuropathic pain alters responses in CNS circuits to mechanical (brush) and thermal (cold and heat) stimuli. *J Neurosci* 2006; 26:10646–10657. 17050704 10.1523/JNEUROSCI.2305-06.2006 PMC6674763

[22] Liu J Hao Y Du M Quantitative cerebral blood flow mapping and functional connectivity of postherpetic neuralgia pain: a perfusion fMRI study. *Pain* 2013; 154:110–118. 23140909 10.1016/j.pain.2012.09.016

[23] Garcia-Larrea L Peyron R Pain matrices and neuropathic pain matrices: a review. *Pain* 2013; 154 suppl 1:S29–S43. 24021862 10.1016/j.pain.2013.09.001

[24] Ostrowsky K Magnin M Ryvlin P Representation of pain and somatic sensation in the human insula: a study of responses to direct electrical cortical stimulation. *Cereb Cortex* 2002; 12:376–385. 11884353 10.1093/cercor/12.4.376

[25] Craig AD A new view of pain as a homeostatic emotion. *Trends Neurosci* 2003; 26:303–307. 12798599 10.1016/s0166-2236(03)00123-1

[26] Kucyi A Salomons TV Davis KD Mind wandering away from pain dynamically engages antinociceptive and default mode brain networks. *Proc Natl Acad Sci USA* 2013; 110:18692–18697. 24167282 10.1073/pnas.1312902110 PMC3832014

[27] Kulkarni B Bentley DE Elliott R Attention to pain localization and unpleasantness discriminates the functions of the medial and lateral pain systems. *Eur J Neurosci* 2005; 21:3133–3142. 15978022 10.1111/j.1460-9568.2005.04098.x

[28] Ichesco E Quintero A Clauw DJ Altered functional connectivity between the insula and the cingulate cortex in patients with temporomandibular disorder: a pilot study. *Headache* 2012; 52:441–454. 21929661 10.1111/j.1526-4610.2011.01998.x PMC3256286

[29] Devinsky O Morrell MJ Vogt BA Contributions of anterior cingulate cortex to behaviour. *Brain* 1995; 118 (pt 1):279–306. 7895011 10.1093/brain/118.1.279

[30] Becerra L Schwartzman RJ Kiefer RT CNS measures of pain responses pre- and post-anesthetic ketamine in a patient with complex regional pain syndrome. *Pain Med* 2015; 16:2368–2385. 26745152 10.1111/pme.12939

[31] Raichle ME The brain's default mode network. *Annu Rev Neurosci* 2015; 38:433–447. 25938726 10.1146/annurev-neuro-071013-014030

[32] Zhang SS Wu W Liu ZP Altered regional homogeneity in experimentally induced low back pain: a resting-state fMRI study. *J Neuroeng Rehab* 2014; 11:115. 10.1186/1743-0003-11-115 PMC4237877 25080831

[33] Kucyi A Moayedi M Weissman-Fogel I Enhanced medial prefrontal-default mode network functional connectivity in chronic pain and its association with pain rumination. *J Neurosci* 2014; 34:3969–3975. 24623774 10.1523/JNEUROSCI.5055-13.2014 PMC6705280

[34] Loggia ML Kim J Gollub RL Default mode network connectivity encodes clinical pain: an arterial spin labeling study. *Pain* 2013; 154:24–33. 23111164 10.1016/j.pain.2012.07.029 PMC3534957

[35] Tessitore A Russo A Giordano A Disrupted default mode network connectivity in migraine without aura. *J Headache Pain* 2013; 14:89. 24207164 10.1186/1129-2377-14-89 PMC3832236

[36] Xue T Yuan K Zhao L Intrinsic brain network abnormalities in migraines without aura revealed in resting-state fMRI. *PLoS One* 2012; 7:e52927. 23285228 10.1371/journal.pone.0052927 PMC3532057

[37] Peelen MV Downing PE Within-subject reproducibility of category-specific visual activation with functional MRI. *Hum Brain Mapp* 2005; 25:402–408. 15852382 10.1002/hbm.20116 PMC6871698

[38] Grill-Spector K Knouf N Kanwisher N The fusiform face area subserves face perception, not generic within-category identification. *Nat Neurosci* 2004; 7:555–562. 15077112 10.1038/nn1224

[39] Parise M Kubo TT Doring TM Cuneus and fusiform cortices thickness is reduced in trigeminal neuralgia. *J Headache Pain* 2014; 15:17. 24661349 10.1186/1129-2377-15-17 PMC3997919

[40] Buvanendran A Ali A Stoub TR The use of brain positron emission tomography to identify sites of postoperative pain processing with and without epidural analgesia. *Anesth Analg* 2007; 105:1784–1786.Table of contents. 18042883 10.1213/01.ane.0000270206.30333.cb

[41] Shimo K Ueno T Younger J Visualization of painful experiences believed to trigger the activation of affective and emotional brain regions in subjects with low back pain. *PLoS One* 2011; 6:e26681. 22073183 10.1371/journal.pone.0026681 PMC3206847

[42] Rocca MA Messina R Colombo B Structural brain MRI abnormalities in pediatric patients with migraine. *J Neurol* 2014; 261:350–357. 24305994 10.1007/s00415-013-7201-y

[43] Wang JJ Chen X Sah SK Amplitude of low-frequency fluctuation (ALFF) and fractional ALFF in migraine patients: a resting-state functional MRI study. *Clin Radiol* 2016; 71:558–564. 27055741 10.1016/j.crad.2016.03.004

[44] Zhao L Liu J Yan X Abnormal brain activity changes in patients with migraine: a short-term longitudinal study. *J Clin Neurol (Seoul Korea)* 2014; 10:229–235. 10.3988/jcn.2014.10.3.229 PMC4101100 25045376

[45] Zhao L Liu J Dong X Alterations in regional homogeneity assessed by fMRI in patients with migraine without aura stratified by disease duration. *J Headache Pain* 2013; 14:85. 24134520 10.1186/1129-2377-14-85 PMC3853130

